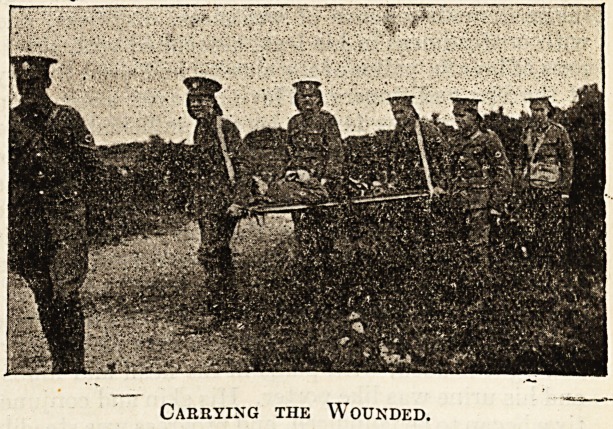# The Medical Unit of the O.T.C.

**Published:** 1910-08-20

**Authors:** 


### The Royal Army Medical Corps Section.

### THE MEDICAL UNIT OF THE O.T.C.

BY A MEMBER OF THE UNIT.

The medical unit of the University of London Officers' Training Corps returned from camp at Aldershot on Saturday last. The students of the various London medical schools who comprise this unit were under the command of Major Herringham, R.A.M.C. (T.), and had as instructors Lieut.-Colonel James and Captain A. C. H. Gray, R.A.M.C.

A Water-cart.

Aldershot is undoubtedly the best place for a unit such as this, for not only are the depôt of the R.A.M.C. and the sanitary school close by, but the Cambridge and Connaught Hospitals afforded some of the senior cadets an opportunity of getting an insight into the organisation of a military hospital. Visits to these latter were paid in the intervals between work in the field, where the unit formed two sections of a field ambulance and provided its own drivers for the fourteen vehicles that form the transport of these sections.

Ambulance Waggons.

There is probably no more complete change for the Londoner than a fortnight under canvas, and no better holiday can be got provided the weather is fine. On this occasion twenty-four hours of a soaking downpour was experienced, but the discomforts were soon forgotten in the sun of the next two days; and the rain had its instructional value, for it made everyone realise more nearly what the work would be like under actual conditions instead of in the luxury of a standing camp in fine weather. The few snapshots our limited space allows us to publish will give some idea of the conditions of camp life during the week.

Sanitation in a camp is one of the most important duties of the R.A.M.C., and in the Officers' Training Corps camp the entire work was left to the

Field Ambulance on the March.

medical unit itself to carry out. In this way much valuable practical knowledge was gained. It is one thing to hear about the disposal of refuse, and to build incinerators that are never to be used; it is quite another to make an incinerator which will

Carrying the Wounded.

destroy all the refuse of a camp and to keep it working in spite of rain and mist.

Altogether, the camp was a great success, whether considered from the advantage to the men's health or the knowledge gained from their stay in camp. It seems a pity that every medical student cannot be made to go through such a training, and we feel sure that those who have done so will never regret it.

## Figures and Tables

**Figure f1:**
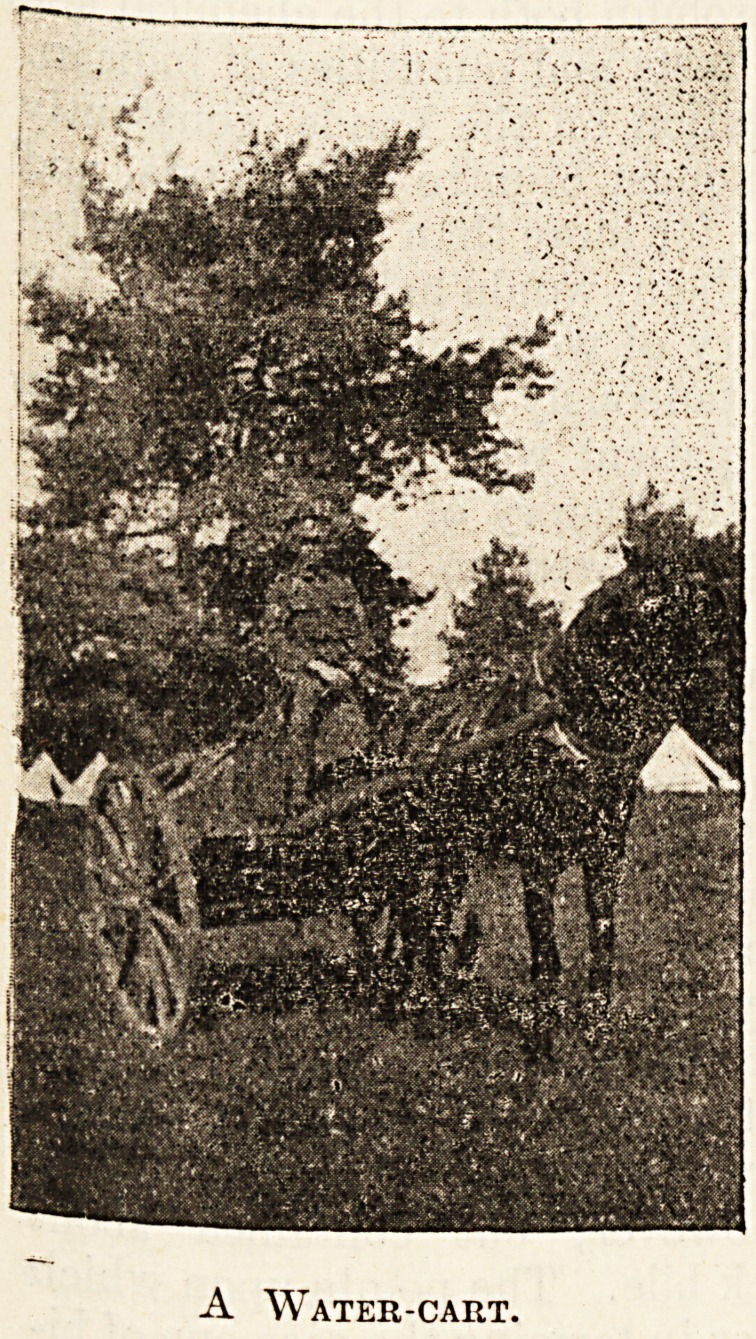


**Figure f2:**
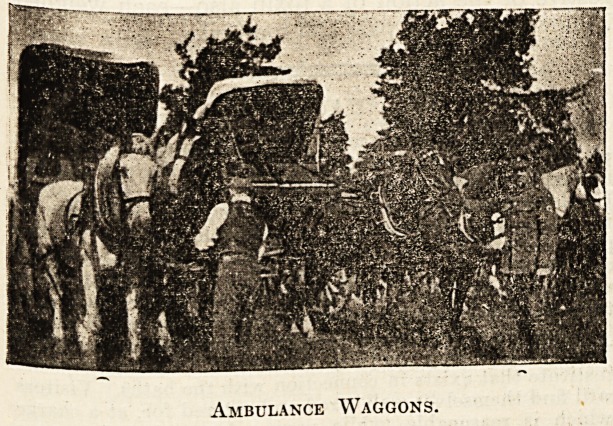


**Figure f3:**
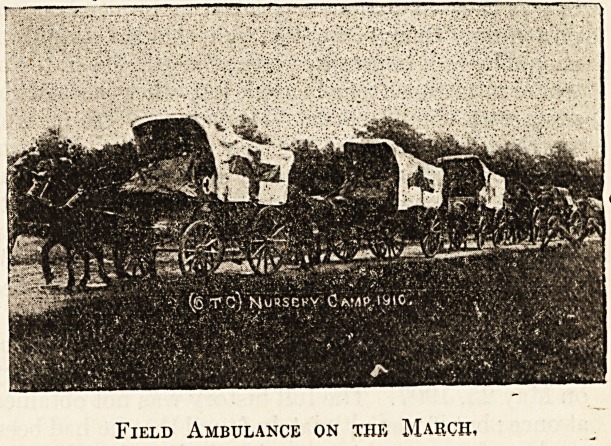


**Figure f4:**